# Superfruits in China: Bioactive phytochemicals and their potential health benefits – A Review

**DOI:** 10.1002/fsn3.2614

**Published:** 2021-10-03

**Authors:** Jinfang Liu, Duoxia Xu, Shuai Chen, Fang Yuan, Like Mao, Yanxiang Gao

**Affiliations:** ^1^ Beijing Laboratory for Food Quality and Safety, Beijing Key Laboratory of Functional Food from Plant Resources Key Laboratory of Healthy Beverages China National Light Industry College of Food Science & Nutritional Engineering China Agricultural University Beijing China; ^2^ Beijing Engineering and Technology Research Center of Food Additives Beijing Technology & Business University Beijing China

**Keywords:** antioxidants, health benefits, phytochemicals, superfruits

## Abstract

The term “superfruit” usually refers to certain fruits, which are rich in antioxidant components, therefore, are beneficial to human health. In China, there has been the concept of health preservation and dietary therapy through food intake in a long history. However, some other superfruits growing mainly in China have not attracted extensive attention, such as Cili, Goji berry, and sea buckthorn. Many studies suggested all of these superfruits showed strong antioxidant effects and anti‐inflammatory activity in common. However, there are various other advantages and functions in different fruits. This article reviewed the research findings from the existing literature published about major antioxidant bioactive compounds and the potential health benefits of these fruits. The phytochemicals from superfruits are bioaccessible and bioavailable in humans with promising health benefits. More studies are needed to validate the health benefits of these superfruits. It would provide essential information for further research and functional food development.

## Graphical abstract

This paper reviewed the recent studies on superfruits in China and emphasized their valuable or unique bioactive components and their nutritional and medicinal values.

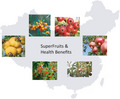

## 1 INTRODUCTION

It is widely known that nutrient intake is the most important mean of maintaining health and preventing diseases. Since the use of some synthetic antioxidants has been restricted for their possible toxic and carcinogenic effects (Yen et al., 1998), food containing natural antioxidants gained worldwide popularity. Nutritional studies paid much attention to vegetables and fruits for their roles in alleviating the risk of numerous non‐communicable diseases (Shahidi & Ambigaipalan, 2015). Regular consumption of fruits and vegetables has been demonstrated to be associated with a reduced risk of certain chronic diseases due to the presence of phytochemicals with antioxidant activities (Zhu et al., 2019).

**FIGURE 1:**
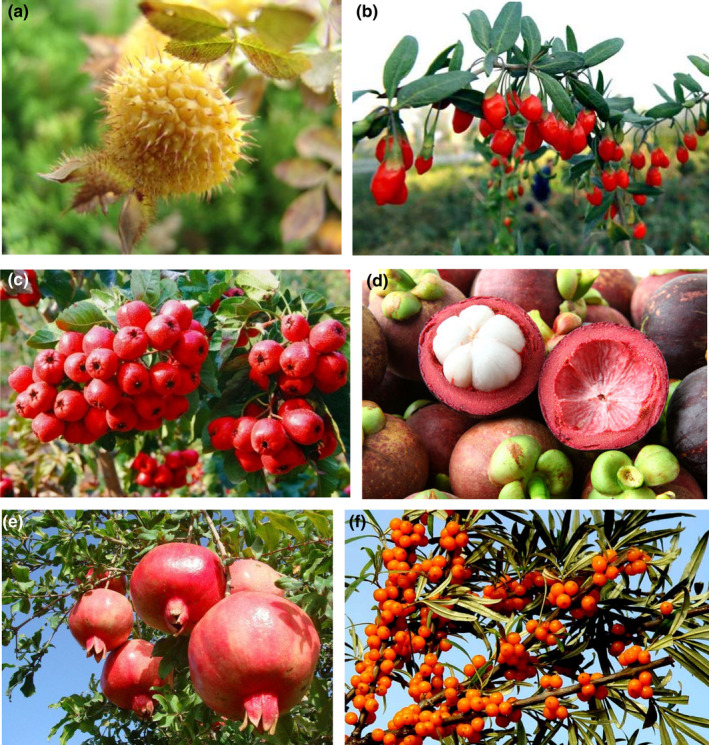
Pictures of the Superfruits: (a) Cili; (b) Goji berry; (c) Hawthorn; (d) Mangosteen; (e) Pomegranate; (f) Sea buckthorn

‘Superfruit’ is a term for fruit with supposed health benefits or therapeutic value as a result of some parts of its nutritional analysis or its overall nutrient density. Usually, fruits that have been recognized as superfruits are antioxidant‐rich. For instance, blueberry is known as a superfruit due to its powerful antioxidant property (Davidson et al., 2018). In recent years, some other fruits with extremely high contents of antioxidants are also called “superfruits,” such as açaí, acerola, goji berry, and mangosteen (Felzenszwalb et al., 2013; Oliveira et al., 2012; Pedro et al., 2018; Prakash & Baskaran, 2018; Wittenauer et al., 2016). The health benefits and potential applications of superfruits could be better exploited if more research is available (De Souza Sant’Ana, 2011). Chang et al. (2019) reviewed the phytochemicals, antioxidant efficacies, and health effects of a list of superfruits (açai, acerola, camu‐camu, goji berry, jaboticaba, jambolao, maqui, noni, and pitanga). However, due to the abundant geographical and climatic conditions, China is a place of production of several superfruits. This review discusses the bioactive ingredients and their potential health benefits of the selected superfruits in China. It is aimed to arouse researchers’ interests in various scientific fields to study superfruits as functional foods or functional food ingredients, and hence, stimulating large‐scale commercial cultivation (Figure 1).

## 2 THE SCIENTIFIC MECHANISM OF PHYTOCHEMICALS WITH ANTIOXIDANT ACTIVITY OF SUPERFRUITS

Oxidative stress releases free oxygen radicals in the body to induce many disorders including cardiovascular malfunction, cancers, cataracts, aging, and other immune diseases (Kaur & Kapoor, 2001; Malik et al., 2005). Antioxidant refers to a compound that can delay or inhibit the oxidation of lipids or other molecules through the initiation or spread of oxidative chain reaction that alleviates the oxidative damage in the human body (Tachakittirungrod et al., 2007). In Table 1, the structural features and antioxidant mechanisms of the major groups in fruits are presented. The antioxidants act as scavengers to neutralize the reactive oxygen species (ROS) by donating one of their own electrons retarding to the electron‐stealing reaction. As the antioxidants are capable to bind metal ions such as copper and iron that catalyze oxidation, they are recognized as chelators as well. Some of the phytochemicals halt cancer by blocking enzymes that enhance cancer or preventing various carcinogens that initiate diseases. There are a number of phytochemicals that could damage cells, tissues, and organelles by producing enzymes that destroy carcinogens in the body and others that suppress the reproduction of cells exposed to carcinogens. Meanwhile, antioxidants are supposed to be beneficial in helping to delay initial episodes of general immune disorders by extending the period between infection and clinical appearance (Kaur & Kapoor, 2001).

**TABLE 1:** Structural features and antioxidant mechanisms of the major groups of fruits (Kalt, 2005Kaur & Kapoor, 2001; Shahidi & Ambigaipalan, 2015)

Antioxidant group	Representative structure	Antioxidant mechanism	Key feature
Ascorbic acid	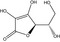	Direct electron donation enzymatic reduction ROS quenching	Vicinal OH groups
Tocopherols	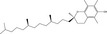	Reacting with lipid peroxyl radicals to produce a tocopheroxyl radical	Conjugated double bonds
Flavonoids	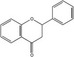	Hydrogen/Electron donation to reduce free radicals Delocalize the unpaired electron leading to the formation of stable phenoxyl radical	Vicinal OH groups Conjugated double bonds
Carotenoids	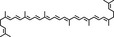	Electron donation ROS quenching	Conjugated double bonds
Phenolics	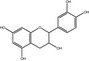	Electron donation metal ion chelation Ascorbic acid sparing ROS quenching	Vicinal OH groups Conjugated double bonds

Note

ROS, reactive oxygen species.

Normally, superfruits are rich in a number of phytochemicals that are of great antioxidative capacity. The most interesting dietary constituents are carotenoids such as β‐carotene, ascorbic acid, tocopherols, dietary fiber, and polyphenols including anthocyanins, flavonols, tannins, and flavonoids (Flores et al., 2012). In general, ascorbic acid is localized in the apoplast, cytosol, mitochondria, vacuole, and plastid; anthocyanins are usually found in fruit peels. Proanthocyanidins are abundant in the peel and especially seeds of berries (Soong & Barlow, 2004). However, the antioxidant activities of different superfruits vary widely based on the assay type, where different assays follow different mechanisms of action and hence may afford different antioxidant activity trends among superfruits. This suggests the need to perform more than one type of antioxidant activity measurement to consider the various mechanisms of antioxidant action and the limitations of each assay. Thus, it is difficult to compare the antioxidant efficacies between different superfruits.

## 3 TYPICAL SUPERFRUITS IN China AND THEIR POTENTIAL HEALTH BENEFITS

Some typical superfruits in China are reviewed in this section. Tables 2 and 3 summarized the main bioactive components and potential health benefits. Various human intervention and animal studies have evaluated the potential health benefits of selected superfruits. All the selected superfruits were reported with antioxidant and anti‐inflammatory activity in common, while some of them reported with unique effects such as hepatoprotective (pomegranate and sea buckthorn), radioprotective (Cili), and vision‐protective (goji berry). The risk of toxicity of the mentioned superfruits as functional foods requires more investigation. Allergic reactions were reported from goji berry consumption. Further studies into the safety and toxicological properties of these superfruits are urgently needed since they might pose allergenic or chemical toxicity risks, especially for people not from China or Asia.

**TABLE 2:** Major antioxidant bioactive compounds in superfruits

	Major antioxidant bioactive compound	Reference
CiLi	Ascorbate Tocopherols Vitamin B_1_ Flavonoids Superoxide dismutase Water‐soluble polysaccharides Organic acids, triterpenes, polysaccharides	Zhang et al. (2001) Wang et al. (2018)
Goji berry	Carotenoids Ascorbic acid Tocopherols Syringic Chlorogenic Gallic Caffeic P‐coumaric 4‐hydroxybenzoic Ferulic Trans‐cinammic Rutin Naringin Quercetin Catechin Kaempferol	Fiorito et al. (2019) Pedro et al. (2018) Amagase and Farnsworth (2011)
Hawthorn	Flavonoids Phenols Oligomeric Procyanidins Chlorogenic Acid Epicatechin Hyperoside Isoquercitrin Rutin Vitexin−4 ''‐O‐Glucoside Vitexin−2 ''‐O‐Rhamnoside, Hyperoside Vitexin Shanyenoside A Quercetin	Chang et al. (2001) (Zhu et al., 2015)
Mangosteen	Tricyclic isoprenylated polyphenols Xanthones Benzophenones Biflavonoid Mangostin Tannin Chrysanthemin Garcinone Gartanin Ascorbic acid	Chen et al. (2019) Gutierrez‐Orozco and Failla (2013) Acuña et al. (2012) Moongkarndi et al. (2004)
Pomegranate	Punicalagin Ellagic acid Anthocyanins Gallotannins Hydroxybenzoic acids Hydroxycinnamic acids Dihydroflavonols	Putnik et al. (2019)
Sea buckthorn	Carotenoids Ascorbic acid Tocopherols Isorhamnetin‐rutinoside Isorhamnetin‐glycoside Quercetin‐rutinoside Quercetin‐glycoside Unsaturated fatty acids	Nelson and Olas (2018) Eccleston et al. (2002)

**TABLE 3:** Potential health benefits of superfruits

	Potential health benefits	Reference
CiLi	Antioxidant Anti‐inflammatory Antioxidant, Antimutagenic Antiatherogenic Antitumor Radioprotective activities	Xu et al. (2018) Wang et al. (2018)
Goji berry	Antioxidant Anti‐inflammatory Vision‐Protective effect Lipid‐Lowering effect Hypoglycaemic effect Anticancer Antitumour Immunostimulatory Neurological Protective effect Modulatory effect Antiaging effect Cardiovascular Protective effect	Ma et al. (2019)
Hawthorn	Anti‐inflammatory Gastroprotective Antimicrobial activities Antioxidant Antithrombotic Anti‐atherosclerotic Treatment of stress, nervousness, sleep disorders, and pain control (antinociceptive) hypotensive, Antihyperlipidemic Antihyperglycemic Anxiolytic Immunomodulatory Antimutagenic	Kumar et al. (2012) Arslan and Bektas (2018) Can et al. (2010) Orhan (2019)
Mangosteen	Antioxidant Antiproliferative Pro‐apoptotic Anti‐inflammatory Maintaining cardiovascular system and gastrointestinal health Anticarcinogenic activities Anticancer Antimicrobial Antidiabetes	Chen et al. (2019) Aizat et al. (2019) Gutierrez‐Orozco and Failla (2013)
Pomegranate	Anticancer Antioxidant Anti‐inflammatory Antidiabetic Antimicrobial Anticarcinogenic Anti‐atherosclerotic Hepatoprotective Neuroprotective activities	Adu‐frimpong et al. (2018) Putnik et al. (2019)
Sea buckthorn	Anti‐inflammatory Anticancer Antioxidant Anti‐atherosclerotic effects Hepatoprotective	Nelson and Olas (2018)

### 3.1 Cili

Cili (*Rosa roxburghii Tratt*) is a kind of specific wild plant in Southwest China. Cili consists of several important components such as superoxide dismutase (SOD), polysaccharide, vitamin C, vitamin E, and some mineral elements (Zn and Ca). Additionally, SOD has long been regarded as a free radical scavenger and ascorbate, which is a highly potent aqueous‐phase antioxidant in plasma (Frei, 1991). This fruit has been known to have a number of beneficial effects on atherosclerosis, cancer, aging, and immunity stress. A set of indices, such as the activity of natural killer (NK) cells, free radical metabolism, microcirculation parameters, cognitive function, light reaction time, and cardiovascular function were selected to evaluate the effects of Cili among 50–75 years old people (30 men and 30 women). It demonstrated that Cili was able to enhance natural killer cell activity and strengthen immune function. Furthermore, the supplementation of Cili would significantly improve the antioxidative capacity and then reduce the injury effect on the endothelium of capillary, artery, and brain with the mechanism probably due to its bioactive components such as SOD, polysaccharides, vitamin C, vitamin E, *etc* ( Ma et al., 1997).

Zhang et al. (2001) studied the mechanism of antiatherogenic effects in cholesterol‐fed animals with Cili juice and they found that the juice not only remarkably reduced low‐density lipoprotein (LDL) oxidative susceptibility but also suppressed oxidized Ox‐LDL‐induced macrophage growth and particularly Ox‐LDL‐induced cholesteryl ester (CE) accumulation in murine peritoneal macrophages by promoting cellular cholesterol efflux. These results indicated that the Cili juice exerted its antiatherogenic effects largely due to its ability to inhibit the oxidative modification of LDL and suppress the formation of foam cells.

Furthermore, the flavonoids of Cili exhibit radioprotection and anti‐apoptosis properties via the Bcl‐2(Ca^2+^)/Caspase‐3/ PARP‐1 pathway in mouse thymus (Xu et al., 2016). In addition, water‐soluble polysaccharide (RTFP) from Cili has shown the potential to be a functional ingredient or hypoglycemic agent in food, pharmaceutical, and cosmetic preparations (Wang et al., 2018). Later, the same research group reported that the digestion properties of a novel polysaccharide from Cili (RTFP‐3) under saliva simulated gastric, and small intestinal conditions were studied. It was proven to be a functional ingredient to improve human health and prevent diseases through regulating gut flora (Wang et al., 2019).

### 3.2 Goji berry

Goji (*Lycium barbarum* L.) berry has been used for centuries in traditional medicine practice in China. It contains mainly polysaccharides, polyphenols, and carotenoids with an ability to exert beneficial effects for the prevention of chronic diseases (cancer, atherosclerosis, obesity, and diabetes), and to promote weight loss and longevity in rats (Amagase & Farnsworth, 2011; Fiorito et al., 2019; Ma et al., 2019; Pedro et al., 2018). Also, there were results that showed that goji berry demonstrated significant reductions in feelings of tiredness after exercise in the human subjects tested. This indicates that goji berry may attenuate stress‐related reactivity and facilitate adaptation to physical stressors during exercise (Amagase & Nance, 2011; Chang et al., 2019). The content of polysaccharides in goji berry is more than 40% (Chan et al., 2007). Polysaccharides purified from goji berry were reported to be effective in various potential health benefits. Wang et al. (2002) found that goji polysaccharides were able to protect the seminiferous epithelium from structural damage and apoptosis, in testicular tissue culture and inhibit lipid peroxidation and cytochrome C suggesting an anti‐inflammatory effect. Zhao et al. (2005) tested the influence of polysaccharides on the expression of matrix‐digesting enzymes as skin cancer and aging were associated with the upregulation of matrix metalloproteinase. These results showed that polysaccharides, especially LbGp5, might have visual skin‐protective properties. As goji has been used for hundreds of years for protecting the eyes in Eastern World, Chan et al. (2007) investigated the therapeutic function of this fruit against neurodegeneration in the retina of the rat OH model. They represented that the fruit extract could benefit neural tissue by inhibiting the loss of retinal ganglion cells in glaucoma. Li, Zhou, et al. (2007) concluded that goji polysaccharides were efficient antioxidants that can protect rat liver mitochondria from irradiation‐induced lipid peroxidation and protein oxidation by augmenting endogenetic antioxidant enzymes. Zhu et al. (2007) illustrated that LBP could elicit phenotypic and functional maturation of murine bone marrow‐derived dendritic cells might result in increasing the antitumor effects of dendritic cell‐based vaccine therapy. Meanwhile, Li, Ma, et al. (2007) observed that the treatment with LBP significantly raised antioxidant enzymes activity and inhibited malondialdehyde formation in the mice's heart, brain, and serum. In addition, Le et al. (2007) reported that 95% aqueous ethanol extract of the fruit contained a great amount of flavonoids, including 247 μg myricetin, 296 μg quercetin, and 135 μg kaempferol. Recently, Jeszka‐Skowron et al. (2018) reported that dried fruit extract prepared from goji showed a significant antioxidation activity as well. However, beyond their beneficial properties, goji berry contains renowned allergenic proteins, and, therefore, deserves inclusion among the allergenic foods capable of inducing allergic reactions in sensitive consumers (Uasuf et al., 2020).

### 3.3 Hawthorn

Hawthorn (*Crataegus pinnatifida*) is a genus of fruit‐bearing trees or shrubs distributed in East Asia, North America, Central Asia, and Europe between 30 and 50° of north latitude, belonging to the *Rosaceae* family. According to the climate, the cultivation, the utilization, and the geographical location in China, they can be roughly divided into five producing areas, with 18 species and 6 varieties planted (Delprete, 1997; Guo et al., 2019). Hawthorns are among the most economically important plant species in China, owing to their pleasant flavor, attractive color, and nutrient‐rich fruit. Also, it is considered a highly important medicinal and aromatic plant that has been used for many years for the treatment of various diseases (Arslan & Bektas, 2018). Chinese hawthorn has been widely used in the treatment of hyperlipidemia and cardiovascular diseases. In folk medicine, hawthorn has been used to treat asthma, hyperlipidemia, heart failure, and in Iran and Mexico, for pain as well (Arslan & Bektas, 2018; Cervantes‐Paz et al., 2018; Kisioglu & Nergiz‐Unal, 2018).

Hawthorn leaves, fruits, and seeds have various active substances such as, flavonoids, triterpenic acids, and sesquiterpenes, which through different mechanisms could be beneficial for humans. Various studies have shown that hawthorn can have beneficial effects on controlling and treating cardiovascular diseases, high blood sugar, dyslipidemia, obesity, and atherosclerosis (Bleske et al., 2008; Chang et al., 2005). Dehghani et al. (2019) reported that flavonoids extracted in the leaves of hawthorn can significantly reduce atherosclerotic lesion areas, the fruit extracts contain two triterpenic acids (oleanolic acid and ursolic acid), that have the ability to inhibit the acyl‐coA‐cholesterol acyltransferase enzyme and as a result reduce very low‐density lipoprotein (VLDL) and LDL cholesterol levels. Also, they reported a sesquiterpene found in the seeds of hawthorn, which exhibits the ability to inhibit platelet aggregation, thus showing antithrombotic activity. In addition, a series of metabolic syndrome effects of hawthorn, such as anti‐diabetic and anti‐obesity by lower plasma glucose and decrease in the rate of gluconeogenesis, anti–hyperlipidemia, and reduced atherosclerosis (in vivo and in vitro studies) were reported (Shahrzad Dehghani et al., 2019).

Hawthorn has a high pectin content compared with other fruits. The hawthorn pectin content in fresh fruit is as high as 6.4% and the pectin oligosaccharides from hawthorn showed potential antiglycation activities (Zhu et al., 2019). Moreover, in vitro antioxidant activity assays indicated that ultrasonic treatment significantly improved the antioxidant activity of pectin ultrasonic treatment and is an effective way to enhance the antioxidant activity. (Chen et al., 2019).

### 3.4 Mangosteen

The fruit of *G. mangostama* L. is commonly known as mangosteen, which is referred to as “the queen of fruits” in Southeast Asia (Acuña et al., 2012; Chin et al., 2008; Moongkarndi et al., 2004). It has been used as a traditional medicine for the treatment of diarrhea, inflammation, ulcer, skin infection, abdominal pain, astringent, dysentery, leucorrhoea, and gonorrhea for many years (Chin et al., 2008; Matsumoto et al., 2004; Moongkarndi et al., 2004). The pericarp of mangosteen contains mangostin, tannin, xanthone, chrysanthemin, garcinone, gartanin, vitamin B_1_, B_2_, C, and other bioactive substances (Gutierrez‐Orozco & Failla, 2013; Moongkarndi et al., 2004). In addition, the pericarp of mangosteen showed potential as antioxidant ingredients in cosmetic formulations (Wittenauer et al., 2016). Xanthones were considered to be really important for chemopreventive or therapeutic functions. Several studies represented that xanthone derivatives, as the major secondary metabolites of mangosteen, demonstrated antibacterial, antifungal, antioxidant, anticancer, antiplasmodial, and cytotoxic activities (Gopalakrishnan et al., 1997; Gutierrez‐Orozco & Failla, 2013; Ji et al., 2007; Nakatani et al., 2002; Sakagami et al., 2005; Suksamrarn et al., 2006; Yu et al., 2007). So far, there are more than 68 xanthones isolated from the mangosteen fruit with the majority of them being α‐ and γ‐mangostin (Aizat et al., 2019).

Jung et al. (2006) found that α‐mangostin, one of the important xanthone derivatives, could inhibit alveolar duct formation in a mouse mammary organ culture model and alleviate the carcinogen‐induced formation of aberrant crypt foci in a short‐term colon carcinogenesis model. Matsumoto et al. (2004) reported a great cytotoxic activity of several xanthones against human leukemia HL60 cells, where α‐mangostin presented the most dramatic activity and induced apoptosis in human leukemia cell lines HL60, K562, NB4, and U937. Sato et al. (2004) illuminated that α‐mangostin‐induced apoptosis through the mitochondrial is associated with the inhibition of the Ca^2+^ ATPase pathway in rat pheochromocytoma PC12 cells. γ‐mangostin is a tetraoxygenated diprenylated xanthone derivative. It has been found that γ‐mangostin was able to bind to cyclooxygenase and inhibit its activity resulting in reduced production of prostaglandin E_2_ (PGE_2_), which would affect the activities of some cell types, such as neurons, glial, and endothelial cells at a high level (Nakatani et al., 2002). The function of γ‐mangostin is supposed to contribute to its anti‐inflammatory activity. Moongkarndi et al. (2004) studied the antiproliferative, apoptotic, and antioxidative properties of crude methanolic extract (CME) from mangosteen. The results implied that the CME decreased the intracellular ROS production on SKBR3 human breast cancer cell lines significantly. The components in mangosteen probably serve as the potent anticancer agents and free radical scavengers. In 2013, Gutierrez‐Orozco and Failla made a review of in *vivo* studies on the bioavailability and metabolism of mangosteen xanthones. More recently, novel xanthones have been discovered such as 1,3,6‐trihydroxy‐2‐(3‐methylbut‐2‐enyl)‐8‐(3‐formyloxy‐3‐methylbutyl)–xanthone, 7‐O‐demethyl mangostin, garmoxanthone as well as mangostanaxanthone III, IV, V, VI, and VII. These xanthones were also implicated in various pharmaceutical properties, but more studies are needed to verify their effectiveness in human applications (Aizat et al., 2019).

### 3.5 Pomegranate

The fruit known as pomegranate (*Punica granatum*) originated from the Middle East, then extended to Mediterranean areas, as well as in countries such as Iran, India, China, Japan, and Russia. Pomegranate has been used as a traditional medicine in Asian cultures to treat different ailments (Adu‐frimpong et al., 2018). Many epidemiological studies of the potential effects of pomegranate on cancer prevention, such as lung cancer (Khan et al., 2007), skin cancer (Rout & Banerjee, 2007), prostate cancer (Malik et al., 2005), breast cancer (Toi et al., 2003), etc. suggested that pomegranate could serve as a possible chemopreventive and therapeutic agent against different cancers. This fruit comprises three parts, the seeds, about 3% of the fruit weight, the juice, about 30% of the weight, and the peels (pericarp). Other parts of the pomegranate including roots, bark, leaves, and flowers are also useful (Lansky & Newman, 2007). Pomegranate juice is reported to have strong antioxidant and anti‐atherosclerotic functions due to its high portion of polyphenols such as ellagic acid (EA) in its free and bound forms (ellagitannins and EA glycosides), gallotannins, anthocyanins (cyaniding, delphinidin, and pelargonidin glycosides), and flavonoids (quercetin, kaempferol, and luteolin glycosides) (Malik et al., 2005; Putnik et al., 2019). Seeram et al. (2005) presented that punicalagin, EA, and total pomegranate tannin could reduce the cell number of human oral, prostate, and colon tumor cells. Furthermore, when concentrations of those compounds rose up to an equivalent level (w/w) with pomegranate juice, they were able to induce apoptosis in HT‐29 cells. Punicalagin is supposed to be the most potent antioxidant ingredient for its antioxidant properties. The radical scavenging ability of punicalagin was because of polyphenolic hydroxyl groups that enhance the antioxidative activity through additional resonance stability and *o*‐quinone or *p*‐quinone formation (Kulkarni et al., 2004). In addition, pomegranate juice consumption resulted in antiatherogenic influence with a remarkable reduction in oxidative stress in serum and monocytes‐macrophages, and macrophage uptake of oxidized LDL and then cellular cholesterol biosynthesis (Fuhrman et al., 2005; Rosenblat, Hayek, & Aviram, 2006). Some studies showed that both pomegranate flower and juice might prevent diabetic sequelae via peroxisome proliferator‐activated receptor‐γ binding and nitric oxide production. Antidiabetic compounds included oleanolic, ursolic, and gallic acids (Katz, Newman, & Lansky, 2007). Pomegranate juice was also reported to decrease the potent downregulation of NOSⅢ induced by the oxidation of LDL in human coronary endothelial cells (Nigris et al., 2007). It has been considered that this fruit juice may be useful in Alzheimer's disease, as supplementation of mice with PJ led to significantly less accumulation of soluble Aβ42 and amyloid deposition in the hippocampus (Hartman et al., 2006).

However, the total content of anthocyanins in pomegranate juice was reported to be higher than any other fruit juice tested for antioxidant activity. Pomegranate juice increased the biological actions of NO by protecting NO against oxidative destruction but reversed proatherogenic effects induced by perturbed shear stress (de Nigris et al., 2007; Ignarro et al., 2006).

The result from Kohno et al. (2004) suggested that administration of pomegranate seed oil (PSO) that was rich in *c*9, *t*11, and *c*13‐CLN could inhibit azoxymethane‐induced colon carcinogenesis, while Yamasaki et al. (2006) found that PSO promoted Ig production by mouse splenocytes. In addition, emerging evidence has suggested that nutraceutical ingredients like PSO possessed health‐promoting effects in cell and animal models. However, these health benefits (anticancer, antioxidant, anti‐inflammatory, anti‐diabetic, and so on) are limited by low physicochemical stability, slow intestinal absorption, and rapid metabolism of PSO (Adu‐frimpong et al., 2018).

### 3.6 Sea buckthorn

Sea buckthorn (*Hippophae rhamnoides* L.) belongs to the *Elaeagnaceae* family, which is naturally distributed throughout Eurasia from the Baltic Sea and the North Sea in the west to Central Asia in the east (Guliyev et al., 2004; Negi et al., 2005; Nelson & Olas, 2018). This fruit is elliptic or oval in shape, and it is a yellowish‐orange berry with silvery dust particles covered surface, and sour in taste (Guliyev et al., 2004). Sea buckthorn consists of series of chemical compounds including vitamins, carotenoids, flavonoids, *etc*. It is found that the juice is rich in vitamin E, vitamin C, and flavonoids that are 13.3, 1,540, and 1,182 mg/L, respectively. More than 75% of the total vitamin E is in the form of α‐tocopherol, and isorhamnetin is one of the most active flavonol aglycones in sea buckthorn juice (Eccleston et al., 2002; Teng et al., 2006). All parts of the plant have been used as a good source of bioactive substances treating diseases in traditional medicine (Geetha et al., 2003). Nowadays, scientific studies have reported pharmacological effects of sea buckthorn. In *vitro* studies and in *vivo* human and animal models, have found that the juices, jams, and oils derived from this fruit and seeds have a wide range of beneficial anti‐inflammatory, anticancer, antioxidant, and anti‐atherosclerotic effects. These were attributed to the presence of phenolics, vitamins, minerals, amino acids, fatty acids, and phytosterols (Nelson & Olas, 2018). Eccleston et al. (2002) demonstrated that supplementation of sea buckthorn juice showed a moderate decrease in the susceptibility of LDL to oxidation and, therefore, its rate of accumulation by macrophages. Teng et al. (2006) studied isorhamnetin, which was the metabolite of quercetin (Ader et al., 2000), and the result illustrated potent cytotoxicity against human hepatocellular carcinoma cells (BEL‐7402). The antioxidant activity of leaf extract of sea buckthorn was also detected by Geetha et al. (2003) studying chromium(VI)‐induced oxidative stress in albino rats. They revealed that the ethanolic leaf extract at a concentration of 100 and 250 mg/kg body weight significantly reduced the chromium‐induced oxidative damage in animals. Meanwhile, methanol extract of sea buckthorn seed exhibited high antibacterial and antioxidant capacity, which is supposed to be due to its high phenolic contents (Negi et al., 2005). Oil extracted from sea buckthorn has high concentrations of lipophilic constituents, predominantly unsaturated fatty acids in triglyceride form, and phytosterols and vitamins A and E have a positive influence on human health, especially on the cardiovascular system (Olas, 2016).

## 4 CONCLUSION

This review has focused on the potential human health benefits of superfruits in China. Evidently, the functional properties are due to their abundant or unique components with high nutrition and medical value. In *vivo* and in *vitro* studies have found that the bioactive phytochemicals present in superfruits have been useful in antioxidant, anti‐inflammatory, and reducing the risk of various diseases, such as heart diseases, various cancers, and brain diseases. Complementary research is also needed to enhance the potential functionalities of the by‐products of these superfruits in China, as such by‐products contain numerous phytochemicals that may be beneficial to human health. In the future, the studies of functional food development of these superfruits could not only benefit the health of consumers but also promote the development of China's fruit industry.

## CONFLICTS OF INTEREST

The authors declare that they have no conflicts of interest.

## References

[fsn32614-bib-0001] Acuña, U. M. , Dastmalchi, K. , Basile, M. J. , & Kennelly, E. J. (2012). Quantitative high‐performance liquid chromatography photo‐diode array (HPLC‐PDA) analysis of benzophenones and biflavonoids in eight Garcinia species. Journal of Food Composition and Analysis, 25(2), 215–220. 10.1016/j.jfca.2011.10.006

[fsn32614-bib-0002] Ader, P. , Wessmann, A. , & Wolffram, S. (2000). Bioavailability and metabolism of the flavonol quercetin in the pig. Free Radical Biology and Medicine, 28(7), 1056–1067. 10.1016/S0891-5849(00)00195-7 10832067

[fsn32614-bib-0003] Adu‐frimpong, M. , Omari‐siaw, E. , & Mukhtar, Y. M. (2018). Formulation of pomegranate seed oil : A promising approach of improving stability and health‐promoting properties. European Journal of Lipid Science and Technology, 1800177, 1–11. 10.1002/ejlt.201800177

[fsn32614-bib-0004] Aizat, W. M. , Jamil, I. N. , Ahmad‐Hashim, F. H. , & Noor, N. M. (2019). Recent updates on metabolite composition and medicinal benefits of mangosteen plant. PeerJ, 7, e6324. 10.7717/peerj.6324 30755827 PMC6368837

[fsn32614-bib-0005] Amagase, H. , & Farnsworth, N. R. (2011). A review of botanical characteristics, phytochemistry, clinical relevance in efficacy and safety of *Lycium barbarum* fruit (Goji). Food Research International, 44(7), 1702–1717. 10.1016/j.foodres.2011.03.027

[fsn32614-bib-0006] Amagase, H. , & Nance, D. M. (2011). Lycium barbarum Increases caloric expenditure and decreases waist circumference in healthy overweight men and women: pilot study. Journal of the American College of Nutrition, 30(5), 304–309. 22081616 10.1080/07315724.2011.10719973

[fsn32614-bib-0007] Arslan, R. , & Bektas, N. (2018). Potential antithrombotic effect of crataegus species. Indian Journal of Pharmaceutical Education and Research, 52(4), S155–S157. 10.5530/ijper.52.4s.93

[fsn32614-bib-0008] Bleske, B. E. , Koch, E. , Aaronson, K. , & Boluyt, M. O. (2008). Hawthorn and heart disease. In R. R. Watson & V. R. Preedy (Eds.), Botanical medicine in clinical practice (pp. 493–502). CABI. Retrieved from http://apps.webofknowledge.com/full_record.do?product=UA&search_mode=GeneralSearch&qid=1&SID=5AfxDMhZDdxlO3CTtrG&page=1&doc=5

[fsn32614-bib-0009] Can, Ö. D. , Özkay, Ü. D. , Öztrk, N. , & Öztürk, Y. (2010). Effects of hawthorn seed and pulp extracts on the central nervous system. Pharmaceutical Biology, 48(8), 924–931. 10.3109/13880200903305500 20673180

[fsn32614-bib-0010] Cervantes‐Paz, B. , Ornelas‐Paz, J. , Gardea‐Bejar, A. , Yahia, E. , Rios‐Velasco, C. , Zamudio‐Flores, P. , & Ibarra‐Junquera, V. (2018). Phenolic compounds of hawthorn (*Crataegus* Spp.): Their biological activity associated to the protection of human health. Revista Fitotecnia Mexicana, 41(3), 339–349.

[fsn32614-bib-0011] Chan, H. C. , Chang, R. C. C. , Koon‐Ching Ip, A. , Chiu, K. , Yuen, W. H. , Zee, S. Y. , & So, K. F. (2007). Neuroprotective effects of *Lycium barbarum* Lynn on protecting retinal ganglion cells in an ocular hypertension model of glaucoma. Experimental Neurology, 203(1), 269–273. 10.1016/j.expneurol.2006.05.031 17045262

[fsn32614-bib-0012] Chang, S. K. , Alasalvar, C. , & Shahidi, F. (2019). Superfruits: Phytochemicals, antioxidant efficacies, and health effects–A comprehensive review. Critical Reviews in Food Science and Nutrition, 59(10), 1580–1604. 10.1080/10408398.2017.1422111 29360387

[fsn32614-bib-0013] Chang, W.‐T. , Dao, J. , & Shao, Z.‐H. (2005). Hawthorn: Potential roles in cardiovascular disease. The American Journal of Chinese Medicine, 33(01), 1–10. 10.1142/S0192415X05002606 15844828

[fsn32614-bib-0014] Chang, Q. , Zhu, M. , Zuo, Z. , Chow, M. , & Ho, W. K. K. (2001). High‐performance liquid chromatographic method for simultaneous determination of hawthorn active components in rat plasma. Journal of Chromatography B, 760, 227–235. 10.1016/S0378-4347(01)00273-0 11530981

[fsn32614-bib-0015] Chen, X. , Qi, Y. , Zhu, C. , & Wang, Q. (2019). Effect of ultrasound on the properties and antioxidant activity of hawthorn pectin. International Journal of Biological Macromolecules, 131, 273–281. 10.1016/j.ijbiomac.2019.03.077 30876895

[fsn32614-bib-0016] Chin, Y. W. , Jung, H. A. , Chai, H. , Keller, W. J. , & Kinghorn, A. D. (2008). Xanthones with quinone reductase‐inducing activity from the fruits of Garcinia mangostana (Mangosteen). Phytochemistry, 69(3), 754–758. 10.1016/j.phytochem.2007.09.023 17991497

[fsn32614-bib-0017] Davidson, K. T. , Zhu, Z. , Balabanov, D. , Zhao, L. , Wakefield, M. R. , Bai, Q. , & Fang, Y. (2018). Beyond conventional medicine ‐ a look at blueberry, a cancer‐fighting superfruit. Pathology and Oncology Research, 24(4), 733–738. 10.1007/s12253-017-0376-2 29285736

[fsn32614-bib-0018] de Souza Sant'Ana, A. (2011). Special issue on exotic fruits. Food Research International, 44(7), 1657. 10.1016/j.foodres.2011.06.017 PMC3156450 21857774

[fsn32614-bib-0019] Dehghani, S. , Mehri, S. , & Hosseinzadeh, H. (2019). The effects of *Crataegus pinnatifida* (Chinese hawthorn) on metabolic syndrome: A review. Iranian Journal of Basic Medical Sciences, 22(5), 460–468. 31217924 10.22038/IJBMS.2019.31964.7678 PMC6556496

[fsn32614-bib-0020] Delprete, P. , & Mabberley, D. J. (1997). The plant‐book: a portable dictionary of the vascular plants. Brittonia, 50(4), 466. 10.2307/2807755

[fsn32614-bib-0021] Denigris, F. , Williamsignarro, S. , Sica, V. , Lerman, L. , Darmiento, F. , Byrns, R. , Casamassimi, A. , Carpentiero, D. , Schiano, C. , & Sumi, D. (2007). Effects of a Pomegranate Fruit Extract rich in punicalagin on oxidation‐sensitive genes and eNOS activity at sites of perturbed shear stress and atherogenesis. Cardiovascular Research, 73(2), 414–423. 10.1016/j.cardiores.2006.08.021 17014835

[fsn32614-bib-0022] Eccleston, C. , Baoru, Y. , Tahvonen, R. , Kallio, H. , Rimbach, G. H. , & Minihane, A. M. (2002). Effects of an antioxidant‐rich juice (sea buckthorn) on risk factors for coronary heart disease in humans. Journal of Nutritional Biochemistry, 13(6), 346–354. 10.1016/S0955-2863(02)00179-1 12088800

[fsn32614-bib-0023] Felzenszwalb, I. , da Costa Marques, M. R. , Mazzei, J. L. , & Aiub, C. A. F. (2013). Toxicological evaluation of Euterpe edulis: A potential superfruit to be considered. Food and Chemical Toxicology, 58, 536–544. 10.1016/j.fct.2013.05.029 23712094

[fsn32614-bib-0024] Fiorito, S. , Preziuso, F. , Epifano, F. , Scotti, L. , Bucciarelli, T. , Taddeo, V. A. , & Genovese, S. (2019). Novel biologically active principles from spinach, goji and quinoa. Food Chemistry, 276, 262–265. 10.1016/j.foodchem.2018.10.018 30409593

[fsn32614-bib-0025] Flores, G. , Dastmalchi, K. , Dabo, A. J. , Whalen, K. , Pedraza‐Peñalosa, P. , Foronjy, R. F. , D’Armiento, J. M. , & Kennelly, E. J. (2012). Antioxidants of therapeutic relevance in COPD from the neotropical blueberry *Anthopterus wardii* . Food Chemistry, 131(1), 119–125. 10.1016/j.foodchem.2011.08.044 22363097 PMC3282679

[fsn32614-bib-0026] Frei, B. (1991). Ascorbic acid protects lipids in human plasma and low‐density lipoprotein against oxidative damage. The American Journal of Clinical Nutrition, 54(6), 1113S–1118S. 10.1093/ajcn/54.6.1113s 1962556

[fsn32614-bib-0027] Fuhrman, B. , Volkova, N. , & Aviram, M. (2005). Pomegranate juice inhibits oxidized LDL uptake and cholesterol biosynthesis in macrophages. Journal of Nutritional Biochemistry, 16(9), 570–576. 10.1016/j.jnutbio.2005.02.009 16115546

[fsn32614-bib-0028] Geetha, S. , Sai Ram, M. , Mongia, S. S. , Singh, V. , Ilavazhagan, G. , & Sawhney, R. C. (2003). Evaluation of antioxidant activity of leaf extract of Seabuckthorn (*Hippophae rhamnoides* L.) on chromium(VI) induced oxidative stress in albino rats. Journal of Ethnopharmacology, 87(2–3), 247–251. 10.1016/S0378-8741(03)00154-5 12860317

[fsn32614-bib-0029] Gopalakrishnan, G. , Banumathi, B. , & Suresh, G. (1997). Evaluation of the antifungal activity of natural xanthones from Garcinia mangostana and their synthetic derivatives. Journal of Natural Products, 60(5), 519–524. 10.1021/np970165u 9213587

[fsn32614-bib-0030] Guliyev, V. B. , Gul, M. , & Yildirim, A. (2004). Hippophae rhamnoides L.: Chromatographic methods to determine chemical composition, use in traditional medicine and pharmacological effects. Journal of Chromatography B: Analytical Technologies in the Biomedical and Life Sciences, 812(1–2), 291–307. 10.1016/j.jchromb.2004.08.047 15556505

[fsn32614-bib-0031] Guo, Q. , Du, J. , Jiang, Y. , Goff, H. D. , & Cui, S. W. (2019). Pectic polysaccharides from hawthorn: Physicochemical and partial structural characterization. Food Hydrocolloids, 90, 146–153. 10.1016/J.FOODHYD.2018.10.011

[fsn32614-bib-0032] Gutierrez‐Orozco, F. , & Failla, M. L. (2013). Biological activities and bioavailability of mangosteen xanthones: A critical review of the current evidence. Nutrients, 5(8), 3163–3183. 10.3390/nu5083163 23945675 PMC3775248

[fsn32614-bib-0033] Hartman, R. E. , Shah, A. , Fagan, A. M. , Schwetye, K. E. , Parsadanian, M. , Schulman, R. N. , Finn, M. B. , & Holtzman, D. M. (2006). Pomegranate juice decreases amyloid load and improves behavior in a mouse model of Alzheimer’s disease. Neurobiology of Disease, 24(3), 506–515. 10.1016/j.nbd.2006.08.006 17010630

[fsn32614-bib-0034] Ignarro, L. J. , Byrns, R. E. , Sumi, D. , de Nigris, F. , & Napoli, C. (2006). Pomegranate juice protects nitric oxide against oxidative destruction and enhances the biological actions of nitric oxide. Nitric Oxide ‐ Biology and Chemistry, 15(2), 93–102. 10.1016/j.niox.2006.03.001 16626982

[fsn32614-bib-0035] Jeszka‐Skowron, M. , Oszust, K. , Zgoła‐Grześkowiak, A. , & Frąc, M. (2018). Quality assessment of goji fruits, cranberries, and raisins using selected markers. European Food Research and Technology, 244(12), 2159–2168. 10.1007/s00217-018-3125-1

[fsn32614-bib-0036] Ji, X. , Avula, B. , & Khan, I. A. (2007). Quantitative and qualitative determination of six xanthones in *Garcinia mangostana* L. by LC–PDA and LC–ESI‐MS. Journal of Pharmaceutical and Biomedical Analysis, 43(4), 1270–1276. 10.1016/j.jpba.2006.10.018 17129697

[fsn32614-bib-0037] Jung, H. A. , Su, B. N. , Keller, W. J. , Mehta, R. G. , & Kinghorn, A. D. (2006). Antioxidant xanthones from the pericarp of *Garcinia mangostana* (Mangosteen). Journal of Agricultural and Food Chemistry, 54(6), 2077–2082. 10.1021/jf052649z 16536578

[fsn32614-bib-0038] Kaur, C. , & Kapoor, H. C. (2001). Antioxidants in fruits and vegetables ‐ the millennium’s health. International Journal of Food Science and Technology, 36(7), 703–725. 10.1046/j.1365-2621.2001.00513.x

[fsn32614-bib-0039] Khan, N. , Hadi, N. , Afaq, F. , Syed, D. N. , Kweon, M.‐H. , & Mukhtar, H. (2007). Pomegranate fruit extract inhibits prosurvival pathways in human A549 lung carcinoma cells and tumor growth in athymic nude mice. Carcinogenesis, 28(1), 163–173. 10.1093/carcin/bgl145 16920736

[fsn32614-bib-0040] Kisioglu, B. , & Nergiz‐Unal, R. (2018). The powerful story against cardiovascular diseases: Dietary factors. Food Reviews International, 34(8), 713–745. 10.1080/87559129.2017.1410172

[fsn32614-bib-0041] Kohno, H. , Suzuki, R. , Yasui, Y. , Hosokawa, M. , Miyashita, K. , & Tanaka, T. (2004). Pomegranate seed oil rich in conjugated linolenic acid suppresses chemically induced colon carcinogenesis in rats. Cancer Science, 95(6), 481–486. 10.1111/j.1349-7006.2004.tb03236.x 15182427 PMC11158596

[fsn32614-bib-0042] Kulkarni, A. P. , Aradhya, S. M. , & Divakar, S. (2004). Isolation and identification of a radical scavenging antioxidant ‐ Punicalagin from pith and carpellary membrane of pomegranate fruit. Food Chemistry, 87(4), 551–557. 10.1016/j.celrep.2014.02.015

[fsn32614-bib-0043] Kumar, D. , Arya, V. , Bhat, Z. A. , Khan, N. A. , & Prasad, D. N. (2012). The genus Crataegus: Chemical and pharmacological perspectives. Brazilian Journal of Pharmacognosy, 22(5), 1187–1200.

[fsn32614-bib-0044] Lansky, E. P. , & Newman, R. A. (2007). Punica granatum (pomegranate) and its potential for prevention and treatment of inflammation and cancer. Journal of Ethnopharmacology, 109(2), 177–206. 10.1016/j.jep.2006.09.006 17157465

[fsn32614-bib-0045] Le, K. , Chiu, F. , & Ng, K. (2007). Identification and quantification of antioxidants in *Fructus lycii* . Food Chemistry, 105(1), 353–363. 10.1016/j.foodchem.2006.11.063

[fsn32614-bib-0046] Li, X. L. , Zhou, A. G. , & Li, X. M. (2007). Inhibition of *Lycium barbarum* polysaccharides and *Ganoderma lucidum* polysaccharides against oxidative injury induced by γ‐irradiation in rat liver mitochondria. Carbohydrate Polymers, 69(1), 172–178. 10.1016/j.carbpol.2006.09.021

[fsn32614-bib-0047] Li, X. M. , Ma, Y. L. , & Liu, X. J. (2007). Effect of the Lycium barbarum polysaccharides on age‐related oxidative stress in aged mice. Journal of Ethnopharmacology, 111(3), 504–511. 10.1016/j.jep.2006.12.024 17224253

[fsn32614-bib-0048] Ma, Z. F. , Zhang, H. , Teh, S. S. , Wang, C. W. , Zhang, Y. , Hayford, F. , & Zhang, Y. (2019). Review article goji berries as a potential natural antioxidant medicine : An insight into their molecular mechanisms of action. Oxidative Medicine and Cellular Longevity, 9. 10.1155/2019/2437397 PMC6343173 30728882

[fsn32614-bib-0049] Malik, A. , Afaq, F. , Sarfaraz, S. , Adhami, V. M. , Syed, D. N. , & Mukhtar, M. (2005). Pomegranate fruit juice for chemoprevention and chemotherapy of prostate cancer. PANS, 102(41), 14813–14818. 10.1016/j.foodchem.2014.12.003 PMC1253570 16192356

[fsn32614-bib-0050] Matsumoto, K. , Akao, Y. , Yi, H. , Ohguchi, K. , Ito, T. , Tanaka, T. , Kobayashi, E. , Iinuma, M. , & Nozawa, Y. (2004). Preferential target is mitochondria in α‐mangostin‐induced apoptosis in human leukemia HL60 cells. Bioorganic and Medicinal Chemistry, 12(22), 5799–5806. 10.1016/j.bmc.2004.08.034 15498656

[fsn32614-bib-0051] Moongkarndi, P. , Kosem, N. , Kaslungka, S. , Luanratana, O. , Pongpan, N. , & Neungton, N. (2004). Antiproliferation, antioxidation and induction of apoptosis by Garcinia mangostana (mangosteen) on SKBR3 human breast cancer cell line. Journal of Ethnopharmacology, 90(1), 161–166. 10.1016/j.jep.2003.09.048 14698525

[fsn32614-bib-0052] Nakatani, K. , Nakahata, N. , Arakawa, T. , Yasuda, H. , & Ohizumi, Y. (2002). Inhibition of cyclooxygenase and prostaglandin E2 synthesis by γ‐mangostin, a xanthone derivative in mangosteen, in C6 rat glioma cells. Biochemical Pharmacology, 63(1), 73–79. 10.1016/S0006-2952(01)00810-3 11754876

[fsn32614-bib-0053] Negi, P. , Chauhan, A. , Sadia, G. , Rohinishree, Y. , & Ramteke, R. (2005). Antioxidant and antibacterial activities of various seabuckthorn ( L.) seed extracts. Food Chemistry, 92(1), 119–124. 10.1016/j.foodchem.2004.07.009

[fsn32614-bib-0054] Nelson, A. , & Olas, B. (2018). The beneficial health aspects of sea buckthorn (*Elaeagnus rhamnoides* ( L .) oil. Journal of Ethnopharmacology, 213, 183–190. 10.1016/j.jep.2017.11.022 29166576

[fsn32614-bib-0055] Olas, B. (2016). Sea buckthorn as a source of important bioactive compounds in cardiovascular diseases. Food and Chemical Toxicology, 97, 199–204. 10.1016/j.fct.2016.09.008 27616182

[fsn32614-bib-0056] Oliveira, L. D. S. , Moura, C. F. H. , De Brito, E. S. , Mamede, R. V. S. , & De Miranda, M. R. A. (2012). Antioxidant metabolism during fruit development of different acerola (*Malpighia emarginata* D.C) clones. Journal of Agricultural and Food Chemistry, 60(32), 7957–7964. 10.1021/jf3005614 22834960

[fsn32614-bib-0057] Orhan, I. E. (2019). Cardiotonic herb. Current Medicinal Chemistry, 25(37), 4854–4865. 10.2174/0929867323666160919095519 27655074

[fsn32614-bib-0058] Pedro, A. C. , Maurer, J. B. B. , Zawadzki‐Baggio, S. F. , Ávila, S. , Maciel, G. M. , & Haminiuk, C. W. I. (2018). Bioactive compounds of organic goji berry (*Lycium barbarum* L.) prevents oxidative deterioration of soybean oil. Industrial Crops and Products, 112, 90–97. 10.1016/j.indcrop.2017.10.052

[fsn32614-bib-0059] Prakash, A. , & Baskaran, R. (2018). Acerola, an untapped functional superfruit: A review on latest frontiers. Journal of Food Science and Technology, 55(9), 3373–3384. 10.1007/s13197-018-3309-5 30150795 PMC6098779

[fsn32614-bib-0060] Putnik, P. , Kresoja, Ž. , Bosiljkov, T. , Režek, A. , Barba, F. J. , Lorenzo, J. M. , Žuntar, I. (2019). Comparing the effects of thermal and non‐thermal technologies on pomegranate juice quality : A review. Food Chemistry, 279, 150–161. 10.1016/j.foodchem.2018.11.131 30611474

[fsn32614-bib-0061] Rout, S. , & Banerjee, R. (2007). Free radical scavenging, anti‐glycation and tyrosinase inhibition properties of a polysaccharide fraction isolated from the rind from *Punica granatum* . Bioresource Technology, 98(16), 3159–3163. 10.1016/j.biortech.2006.10.011 17140791

[fsn32614-bib-0062] Sakagami, Y. , Iinuma, M. , Piyasena, K. G. N. P. , & Dharmaratne, H. R. W. (2005). Antibacterial activity of α‐mangostin against vancomycin resistant Enterococci (VRE) and synergism with antibiotics. Phytomedicine, 12(3), 203–208. 10.1016/j.phymed.2003.09.012 15830842

[fsn32614-bib-0063] Sato, A. , Fujiwara, H. , Oku, H. , Ishiguro, K. , & Ohizumi, Y. (2004). Alpha‐mangostin induces Ca2+‐ATPase‐dependent apoptosis via mitochondrial pathway in PC12 cells. Journal of Pharmacological Sciences, 95, 33–40. 10.1254/jphs.95.33 15153648

[fsn32614-bib-0064] Seeram, N. , Adams, L. , Henning, S. , Niu, Y. , Zhang, Y. , Nair, M. , & Heber, D. (2005). In vitro antiproliferative, apoptotic and antioxidant activities of punicalagin, ellagic acid and a total pomegranate tannin extract are enhanced in combination with other polyphenols as found in pomegranate juice. The Journal of Nutritional Biochemistry, 16(6), 360–367. 10.1016/j.jnutbio.2005.01.006 15936648

[fsn32614-bib-0065] Shahidi, F. , & Ambigaipalan, P. (2015). Phenolics and polyphenolics in foods, beverages and spices: Antioxidant activity and health effects ‐ A review. Journal of Functional Foods, 18, 820–897. 10.1016/j.jff.2015.06.018

[fsn32614-bib-0066] Soong, Y. Y. , & Barlow, P. J. (2004). Antioxidant activity and phenolic content of selected fruit seeds. Food Chemistry, 88(3), 411–417. 10.1016/j.foodchem.2004.02.003

[fsn32614-bib-0067] Suksamrarn, S. , Komutiban, O. , Ratananukul, P. , Chimnoi, N. , Lartpornmatulee, N. , & Suksamrarn, A. (2006). Cytotoxic prenylated xanthones from the young fruit of *Garcinia mangostana* . Chemical & Pharmaceutical Bulletinharmaceutical Bulletin, 54, 301–305. 10.3390/molecules15107438 16508181

[fsn32614-bib-0068] Tachakittirungrod, S. , Okonogi, S. , & Chowwanapoonpohn, S. (2007). Study on antioxidant activity of certain plants in Thailand: Mechanism of antioxidant action of guava leaf extract. Food Chemistry, 103(2), 381–388. 10.1016/j.foodchem.2006.07.034

[fsn32614-bib-0069] Teng, B. , Lu, Y. , Wang, Z. , Tao, X. , & Wei, D. (2006). In vitro anti‐tumor activity of isorhamnetin isolated from Hippophae rhamnoides L. against BEL‐7402 cells. Pharmacological Research, 54(3), 186–194. 10.1016/j.phrs.2006.04.007 16765054

[fsn32614-bib-0070] Uasuf, C. G. , De Angelis, E. , Guagnano, R. , Pilolli, R. , D’Anna, C. , Villalta, D. , Brusca, I. , & Monaci, L. (2020). Emerging allergens in goji berry superfruit: The identification of new ige binding proteins towards allergic patients’ sera. Biomolecules, 10(5), 1–13. 10.3390/biom10050689 PMC7277879 32365614

[fsn32614-bib-0071] Wang, L. , Li, C. , Huang, Q. , Fu, X. , & Liu, R. H. (2019). In vitro digestibility and prebiotic potential of a novel polysaccharide from *Rosa roxburghii* Tratt fruit. Journal of Functional Foods, 52, 408–417. 10.1016/j.jff.2018.11.021

[fsn32614-bib-0072] Wang, L. Zhang, B. , Xiao, J. , Huang, Q. , Li, C. , & Fu, X. (2018). Physicochemical, functional, and biological properties of water‐soluble polysaccharides from *Rosa roxburghii* Tratt fruit. Food Chemistry, 249, 127–135. 10.1016/j.foodchem.2018.01.011 29407915

[fsn32614-bib-0073] Wang, Y. , Zhao, H. , Sheng, X. , Gambino, P. E. , Costello, B. , & Bojanowski, K. (2002). Protective effect of Fructus Lycii polysaccharides against time and hyperthermia‐induced damage in cultured seminiferous epithelium. Journal of Ethnopharmacology, 82(2–3), 169–175. 10.1016/S0378-8741(02)00169-1 12241992

[fsn32614-bib-0074] Wittenauer, J. , Schweiggert‐Weisz, U. , & Carle, R. (2016). In vitro‐study of antioxidant extracts from Garcinia mangostana pericarp and Riesling grape pomace ‐ a contribution to by‐products valorization as cosmetic ingredients. Journal of Applied Botany and Food Quality, 89, 249–257. 10.5073/JABFQ.2016.089.032

[fsn32614-bib-0075] Xu, P. , Cai, X. , Zhang, W. , Li, Y. , Qiu, P. , Lu, D. , & He, X. (2016). Flavonoids of Rosa roxburghii Tratt exhibit radioprotection and anti‐apoptosis properties via the Bcl‐2 ( Ca 2 + )/ Caspase‐3 / PARP‐1 pathway. Apoptosis, 21(10), 1125–1143. 10.1007/s10495-016-1270-1 27401922

[fsn32614-bib-0076] Xu, S.‐J. , Zhang, F. , Wang, L.‐J. , Hao, M.‐H. , Yang, X. , Li, N. , Ji, H. L. , & Xu, P. (2018). Flavonoids of *Rosa roxburghii* Tratt offers protection against radiation induced apoptosis and inflammation in mouse thymus. Apoptosis, 23(9–10), 470–483. 10.1007/s10495-018-1466-7 29995207

[fsn32614-bib-0077] Yamasaki, M. , Kitagawa, T. , Koyanagi, N. , Chujo, H. , Maeda, H. , Kohno‐Murase, J. , Imamura, J. , Tachibana, H. , & Yamada, K. (2006). Dietary effect of pomegranate seed oil on immune function and lipid metabolism in mice. Nutrition, 22(1), 54–59. 10.1016/j.nut.2005.03.009 16226015

[fsn32614-bib-0078] Yen, G. , Chen, H. , & Duh, P. (1998). Extraction and Identification of an Antioxidative Component from Jue Ming Zi (*Cassia tora* L.). Analysis, 8561(97), 820–824. 10.3233/978-1-60750-928-8-1514

[fsn32614-bib-0079] Yong‐Xing, M. A. , Yue, Z. , Chuan‐Fu, W. , Zan‐Shun, W. , Su‐Ying, C. , Mei‐Hua, S. , Jie‐Ming, G. , Jian‐Gang, Z. , Qi, G. U. , & Lin, H. E. (1997). The aging retarding effect of “Long‐Life CiLi”. Mechanisms of Ageing and Development, 96(1–3), 171–180. 10.1016/S0047-6374(97)01890-3 9223119

[fsn32614-bib-0080] Yu, L. , Zhao, M. , Yang, B. , Zhao, Q. , & Jiang, Y. (2007). Phenolics from hull of Garcinia mangostana fruit and their antioxidant activities. Food Chemistry, 104(1), 176–181. 10.1016/j.foodchem.2006.11.018

[fsn32614-bib-0081] Zhang, C. , Liu, X. , Qiang, H. , Li, K. , Wang, J. , Chen, D. , & Zhuang, Y. (2001). Inhibitory effects of rosa roxburghii tratt juice on in vitro oxidative modification of low density lipoprotein and on the macrophage growth and cellular cholesteryl ester accumulation induced by oxidized low density lipoprotein. Clinica Chimica Acta, 313(1–2), 37–43. 10.1016/S0009-8981(01)00647-7 11694237

[fsn32614-bib-0082] Zhao, H. , Alexeev, A. , Chang, E. , Greenburg, G. , & Bojanowski, K. (2005). Lycium barbarum glycoconjugates: Effect on human skin and cultured dermal fibroblasts. Phytomedicine, 12(1–2), 131–137. 10.1016/j.phymed.2003.08.002 15693720

[fsn32614-bib-0083] Zhu, S. , Yan, H. , Niu, K. , & Zhang, S. (2015). Simultaneous determination of seven components from hawthorn leaves flavonoids in rat plasma by LC‐MS/MS. Journal of Chromatographic Science, 53(6), 909–914. 10.1093/chromsci/bmu143 25368407

[fsn32614-bib-0084] Zhu, R. , Zhang, X. , Wang, Y. U. , Zhang, L. , Wang, C. , Hu, F. , Ning, C. , & Chen, G. (2019). Pectin oligosaccharides from hawthorn (Crataegus pinnatifida Bunge. Var. major): Molecular characterization and potential antiglycation activities. Food Chemistry, 286, 129–135. 10.1016/j.foodchem.2019.01.215 30827585

